# Joint production of IL-22 participates in the initial phase of antigen-induced arthritis through IL-1β production

**DOI:** 10.1186/s13075-015-0759-2

**Published:** 2015-09-02

**Authors:** Larissa G. Pinto, Jhimmy Talbot, Raphael S. Peres, Rafael F. Franca, Sérgio H. Ferreira, Bernhard Ryffel, José Carlos F. Aves-Filho, Florêncio Figueiredo, Thiago M. Cunha, Fernando Q. Cunha

**Affiliations:** Department of Pharmacology, Ribeirão Preto Medical School, University of São Paulo, Avenida Bandeirantes, 3900, Ribeirão Preto, São Paulo 14049-900 Brazil; Université d’Orléans and CNRS, UMR 7355 Molecular and Experimental Immunology and Neurogenetics, 3b rue de la Férollerie, 45071 Orléans, France; Laboratory of Pathology, School of Medicine, University of Brasilia, Campus Universitário Darcy Ribeiro, Brasilia, 70910-900 Brazil; Present Address: Aggeu Magalhaes Research Center, Oswaldo Cruz Foundation - FIOCRUZ, Avenida Profesor Moreaes Rego s/n, Recife, 50740-465 Brazil

## Abstract

**Introduction:**

Rheumatoid arthritis (RA) is a chronic autoimmune disease characterized by neutrophil articular infiltration, joint pain and the progressive destruction of cartilage and bone. IL-22 is a key effector molecule that plays a critical role in autoimmune diseases. However, the function of IL-22 in the pathogenesis of RA remains controversial. In this study, we investigated the role of IL-22 in the early phase of antigen-induced arthritis (AIA) in mice.

**Methods:**

AIA was induced in C57BL/6, IL-22^−/−^, ASC^−/−^ and IL-1R1^−/−^ immunized mice challenged intra-articularly with methylated bovine serum albumin (mBSA). Expression of IL-22 in synovial membranes was determined by RT-PCR. Articular hypernociception was evaluated using an electronic von Frey. Neutrophil recruitment and histopathological analyses were assessed in inflamed knee joint. Joint levels of inflammatory mediators and mBSA-specific IgG concentration in the serum were measured by ELISA.

**Results:**

The IL-22 mRNA expression and protein levels in synovial tissue were increased during the onset of AIA. In addition, pharmacological inhibition (anti-IL-22 antibody) and genetic deficiency (IL-22^−/−^ mice) reduced articular pain and neutrophil migration in arthritic mice. Consistent with these findings, recombinant IL-22 joint administration promoted articular inflammation per se in WT mice, restoring joint nociception and neutrophil infiltration in IL-22^−/−^ mice. Moreover, IL-22-deficient mice showed reduced synovitis (inflammatory cell influx) and lower joint IL-1β levels, whereas the production of IL-17, MCP-1/CCL2, and KC/CXCL1 and the humoral immune response were similar, compared with WT mice. Corroborating these results, the exogenous administration of IL-22 into the joints induced IL-1β production in WT mice and reestablished IL-1β production in IL-22^−/−^ mice challenged with mBSA. Additionally, IL-1R1^−/−^ mice showed attenuated inflammatory features induced by mBSA or IL-22 challenge. Articular nociception and neutrophil migration induced by IL-22 were also reduced in ASC^−/−^ mice.

**Conclusions:**

These results suggest that IL-22 plays a pro-inflammatory/pathogenic role in the onset of AIA through an ASC-dependent stimulation of IL-1β production.

**Electronic supplementary material:**

The online version of this article (doi:10.1186/s13075-015-0759-2) contains supplementary material, which is available to authorized users.

## Introduction

Rheumatoid arthritis (RA) is a chronic autoimmune disorder that is characterized by symmetric inflammation of the joints, which leads to the progressive destruction of cartilage and bone [1]. The underlying cause of RA is unknown; however, it is mediated by the persistent production of pro-inflammatory cytokines, matrix metalloproteinases (MMPs) and others mediators, all of which play a key role in triggering synovial cell activation that leads to joint destruction and, consequently, articular pain [2, 3].

Pro-inflammatory cytokines, including tumor necrosis factor (TNF)-α, interleukin (IL)-1β, IL-6 and, more recently, IL-17, play a crucial role in the pathogenesis of arthritis, increasing the recruitment of neutrophils into the joint and driving the enhancement of chemokines and degradative enzymes production [4, 5]. In addition, several groups, including ours, have demonstrated the participation of these cytokines in the development of articular pain, which can act directly or indirectly on nociceptive neurons inducing their sensitization [6–10]. Although the pathogenic effects of these cytokines are well explored, the contribution of IL-22 in this context is not yet fully understood.

IL-22 is an IL-10 family cytokine member produced by several different cell types, including T helper (Th)17 cells, natural killer (NK) cells, γδT cells, Th22 cells and lymphoid tissue inducer-like cells (LTi) [11, 12]. IL-22 acts through a transmembrane receptor complex (IL-22R) comprising the IL-22R1 and IL-10R2 subunits [13, 14]. This heterodimeric receptor is expressed in resident tissue cells and is not expressed by hematopoietic immune cells [15, 16]. Interestingly, because immune cells do not express IL-22R1, IL-22 does not directly regulate the functions of these cells. This fact discriminates IL-22 from the majority of conventional cytokines, which directly act on hematopoietic cells. Of note, a few types of tissue cells express the IL-22R1 chain such as cells of the skin, kidney, and liver, those from the respiratory and digestive system, and those of the joints (synovial fibroblasts), whereas the IL-10R2 subunit is ubiquitously expressed [15]. Thus, the expression of the IL-22R1 chain determines whether a cell is an IL-22 target [15, 17]. IL-22 has many functions such as regulating inflammation and autoimmunity [18–21]. Several studies indicated that IL-22 production is increased during autoimmune diseases, including rheumatoid arthritis [22, 23]. However, the role of this cytokine in the onset of these diseases remains controversial. On the one hand, there is evidence that IL-22 expression in synovial tissue is increased in patients with RA and that its upregulation often correlates with disease activity [24, 25]. Moreover, in experimental models of arthritis, IL-22^−/−^ mice were less susceptible to collagen-induced arthritis (CIA) [26]. On the other hand, there is evidence that IL-22 has an anti-inflammatory effect during CIA through an increased IL-10 response, indicating that IL-22 would have dual effects depending on the phase of the disease [27]. Taking into account these apparent contradictions in the present study, we investigated the contribution of IL-22 and the mechanism underlying the pathogenesis of joint inflammation during the acute phase of antigen-induced arthritis (AIA).

## Methods

### Animals

The experiments were performed using male C57BL/6 wild-type (WT) mice and IL-22, IL-1R1, apoptosis-associated speck-like protein containing a C-terminal caspase recruitment domain (ASC) and Toll-like receptor 4 (TLR4) (all in C57BL/6 background) deficient ^(−/−)^ mice weighing 20–25 g. All knockout mice used in this study were co-housed with WT mice for 2 weeks prior to immunization and throughout the period of arthritis induction. IL-22^−/−^ mice did not present any sign of other phenotypes (such as gut diseases) for the duration of the study. The mice were housed in temperature-controlled rooms (22–25 °C) and given water and food ad libitum at the animal facility in the Department of Pharmacology, Ribeirão Preto Medical School, University of São Paulo, Brazil. The mice were taken to the testing room at least 1 h before experiments and were used once. All experiments were conducted in accordance with the prescribed guidelines on experimental animal welfare of the National Institutes of Health and were approved by the Ethics Committee of the Ribeirão Preto Medical School, University of São Paulo.

### Drugs

The following materials were obtained from the indicated sources: recombinant murine IL-22 (rmIL-22; R&D Systems, Minneapolis, MN, USA), anti-IL-22 antibody (α-IL-22) (16-7222-85; eBioscience, San Diego, CA, USA), zymosan, fucoidin, methylated bovine serum albumin (mBSA) and complete Freund adjuvant (CFA; Sigma-Aldrich, St. Louis, MO, USA). The drugs were diluted in sterile saline.

### Induction of experimental arthritis

#### Antigen-induced arthritis (AIA)

The mice were immunized as previously described [28]. Briefly, the mice were sensitized with 500 μg of mBSA in 0.2 ml of an emulsion containing 0.1 ml saline and 0.1 ml CFA (1 mg/ml of *Mycobacterium tuberculosis*) given by subcutaneous (s.c.) injection on day 0. The mice were boosted with the same preparation on day 7. Sham-immunized (SI) mice were given similar injections but without the antigen (mBSA). Twenty-one days after the initial injection, arthritis was induced in the immunized animals by an intra-articular (i.a.) injection of mBSA (30 μg/joint) or IL-22 (0.1, 0.3, 1 or 3 ng/joint) dissolved in 10 μL of saline into the right femur-tibial joint. It is important to mention that previous reports demonstrated that the administration of mBSA in SI mice induced effects similar to those produced by the injection of saline, indicating that, in immunized mice, mBSA induced a specific immune response, thus excluding the possibility of any contamination [9, 29].

### Zymosan-induced arthritis (ZIA)

ZIA was induced using a previously described protocol [30]. In brief, joint inflammation was induced by the i.a. administration of 30 μg of zymosan (from *Saccharomyces cerevisiae*) that was diluted in 10 μL of saline into the right femur-tibial joint. Control mice were injected with 10 μL of saline into the same joint.

### Evaluation of articular hypernociception

The articular hypernociception of the femur-tibial joint was evaluated using a previously described method [9]. In a quiet room, the mice were placed in acrylic cages (12 × 10 × 17 cm high) with a wire grid floor 15–30 min before testing for environmental adaptation. Stimulations were performed only when the animals were quiet, did not display exploratory movements or defecation and were not resting on their paws. In these experiments, an electronic pressure meter, which comprises a hand-held force transducer fitted with a polypropylene tip (IITC Inc., Life Science Instruments, Woodland Hills, CA, USA), was used. For this model, a large tip (4.15 mm^2^) was adapted to the probe. The investigator (blind to group allocation) was trained to apply the tip perpendicularly to the central area of the plantar surface of the hind paw to induce flexion of the femur-tibial joint, followed by paw withdrawal. A gradual increase in pressure was manually performed in blind experiments. The upper limit pressure was 15 g. The electronic pressure meter apparatus automatically recorded the intensity of the force applied when the paw was withdrawn. The test was repeated until three subsequent consistent measurements (i.e., the variation among these measurements was less than 1 g) were obtained. The flexion-elicited mechanical threshold was expressed in grams (g).

### In vivo neutrophil migration

Immunized or non-immunized mice were challenged with mBSA, IL-22, zymosan or saline directly into the articular cavity. At various time points after injection of the inflammatory stimuli, the mice were sacrificed. The articular cavities were washed twice with 5 μL phosphate-buffered saline (PBS) containing 1 mM ethylenediaminetetraacetic acid (EDTA) and were then diluted to a final volume of 50 μL with PBS/EDTA to evaluate leukocyte migration at the indicated times. The total number of leukocytes was counted in a Neubauer chamber diluted in Turk’s solution. Differential cell counts were determined in cytocentrifuge Rosenfeld-stained slices (Cytospin 4; Shandon, Pittsburgh, PA, USA). Differential cell counts were performed with a light microscope, and the results were expressed as the number (mean ± SEM) of neutrophils per joint cavity.

### Cytokine measurements

At the indicated times after i.a. injection of the inflammatory stimuli, the animals were terminally anesthetized, and the knee joints or synovial membranes were removed and homogenized in 500 μl or 100 μl, respectively, of buffer-containing protease inhibitors. IL-17, IL-1β, IL-22, monocyte chemoattractant protein-1 (MCP-1/CCL2) and keratinocyte-derived chemokine (KC/CXCL1) concentrations were measured by enzyme-linked immunosorbent assay (ELISA) using commercial kits (DuoSet; R&D Systems) as previously described [31]. The results are expressed as pg/joint of each cytokine. As a control, the concentrations of these cytokines were measured in immunized animals injected with saline and in SI mice injected with mBSA.

### Pharmacological treatments

In this study the following pharmacological treatments were used: (i) C57BL/6 immunized mice were co-treated with an antibody against IL-22 (α-IL-22, 5 μg/joint) or control isotype antibody (α-CTL, 5 μg/joint), which was administered simultaneously with mBSA (30 μg/joint) or saline (10 μl) into the right femur-tibial joint, and mechanical articular hypernociception was evaluated 1–7 h after administration of the stimulus, followed by the evaluation of neutrophil migration. (ii) WT or IL-22^−/−^ immunized mice were challenged i.a. with saline or mBSA (30 μg/ joint) and co-treated with a co-injection of rmIL-22 (0.3 ng/joint) or vehicle, and articular hypernociception and neutrophil migration were evaluated 7 h following the challenge. In another set of experiments, mBSA-immunized WT or IL-22^−/−^ mice were injected i.a. with mBSA (30 μg) or saline and co-treated with a co-injection of IL-22 (0.3 ng) or vehicle. The concentrations of IL-1β were determined 3 h after the challenge. (iii) mBSA-immunized mice were pretreated with fucoidin, a leukocyte adhesion inhibitor (20 mg/kg, i.v. 15 min prior to the i.a. injection of IL-22), and levels of IL-1β in the joint were determined by ELISA 3 h after the injection of rmIL-22.

### Anti-mBSA antibody titer measurement

Serum anti-mBSA antibody titers in pooled sera from WT and IL-22^−/−^ mice were measured by ELISA. In brief, 96-well plates were coated with 50 μl mBSA solution (10 μg/ml, in 0.1 M phosphate buffer) overnight at 4 °C. After that, serial dilutions of sera were added and incubated overnight at 4 °C. Bound total immunoglobulin (Ig)G and IgG2a were detected with biotin-conjugated anti-mouse IgG and anti-mouse IgG2a, respectively (Vector Laboratories, Burlingame, CA, USA). Finally, 50 μl avidin-HRP (1:5000 dilution; Dako, Glostrup, Denmark) was added to each well, and after 30 min, the plates were washed, and the color reagent OPD (200 μg/well; Sigma-Aldrich) was added. After 15 min, the reaction was stopped with 1 M H_2_SO_4_ and the OD was read at 490 nm.

### Reverse transcription-polymerase chain reaction (RT-PCR) assays

IL-22 mRNA expression was measured by RT-PCR as previously described [9]. Briefly, mice were sacrificed 3 h after mBSA injections (i.a.), and the synovial membranes were harvested. Total cellular RNA from synovial membranes was extracted using the TRIzol reagent (Invitrogen Life Technologies Corp., Carlsbad, CA, USA) according to the directions supplied by the manufacturer. The purity of the total RNA was measured with a NanoVue Plus spectrophotometer (GE Healthcare, Little Chalfont, UK). The wavelength absorption ratio (260/280 nm) was between 1.8 and 2.0 for all preparations. cDNA was produced from total RNA by reverse transcription (Superscript II, Gibco Life Technologies, Grand Island, NY, USA). Real-time quantitative PCR mRNA analysis was performed on an ABI Prism 7500 Sequence Detection System using the SYBR-green fluorescence system (Applied Biosystems, Warrington, UK) for the quantification of amplicons. RT-PCR was performed in a 13-μL reaction volume and carried out with the following cycling parameters: initial heating at 95 °C (10 min), followed by 40 cycles of 94 °C (1 min), 56 °C (1 min) and 72 °C (2 min). Melting curve analysis was performed (65–95 °C) to verify the amplification of a single product. Samples with more than one peak were excluded. The data were analyzed according to the comparative cycle threshold (CT) method. The primer pairs for mouse glyceraldehyde 3-phosphate dehydrogenase (GAPDH) and IL-22 were as follows:

**Table:** 

IL-22 forward: 5′- AGC AAA TCA GCT CAG CTC CTG- 3′;	
IL-22 reverse: 5′- CTT CTT CTC GCT CAG ACG CA- 3′;	
GAPDH forward: 5′- CAT CTT CTT GTG CAG TGC CA -3′;	
GAPDH reverse: 5′- CGG CCA AAT CCG TTC AC-3′;	

### Histological analysis

Femur-tibial joints were collected 7 h after challenge with mBSA, fixed in 10 % (vol/vol) buffered formalin and decalcified in a solution of sodium citrate and formic acid for 2–3 weeks. The tissues were then trimmed, dehydrated in graded ethanol, embedded in paraffin, cut into 5-μm-thick frontal sections and stained with hematoxylin and eosin (H&E). Two independent observers that were blinded to the treatment graded the extent of synovitis (inflammatory cell influx and synovial hyperplasia). The histological scoring system used was as follows: none (0), mild (1), moderate (2) or severe (3) synovitis.

### Statistical analysis

Data are reported as the means ± SEM and are representative of two separate experiments. Two-way ANOVA was used to compare the groups and doses at the different times (curves) when the hypernociceptive responses were measured after the stimulus injection. The analyzed factors were the treatments, the time, and the time *versus* treatment interaction. If there was a significant time *versus* treatment interaction, one-way ANOVA followed by Bonferroni’s *t* test was performed for each time. Alternatively, if the responses (hypernociception, neutrophil migration, and cytokine production) were measured only once after the stimulus injection, the differences between responses were evaluated by one-way ANOVA followed by Bonferroni’s *t* test (for three or more groups), comparing all pairs of columns, or by two-tailed Student’s *t* test (for two groups). *P* values less than 0.05 were considered significant. Statistical analysis was performed with GraphPad Prism (GraphPad Software, San Diego, CA, USA).

## Results

### Local production of IL-22 participates in the pathogenesis of AIA in mice

**Fig. 1:**
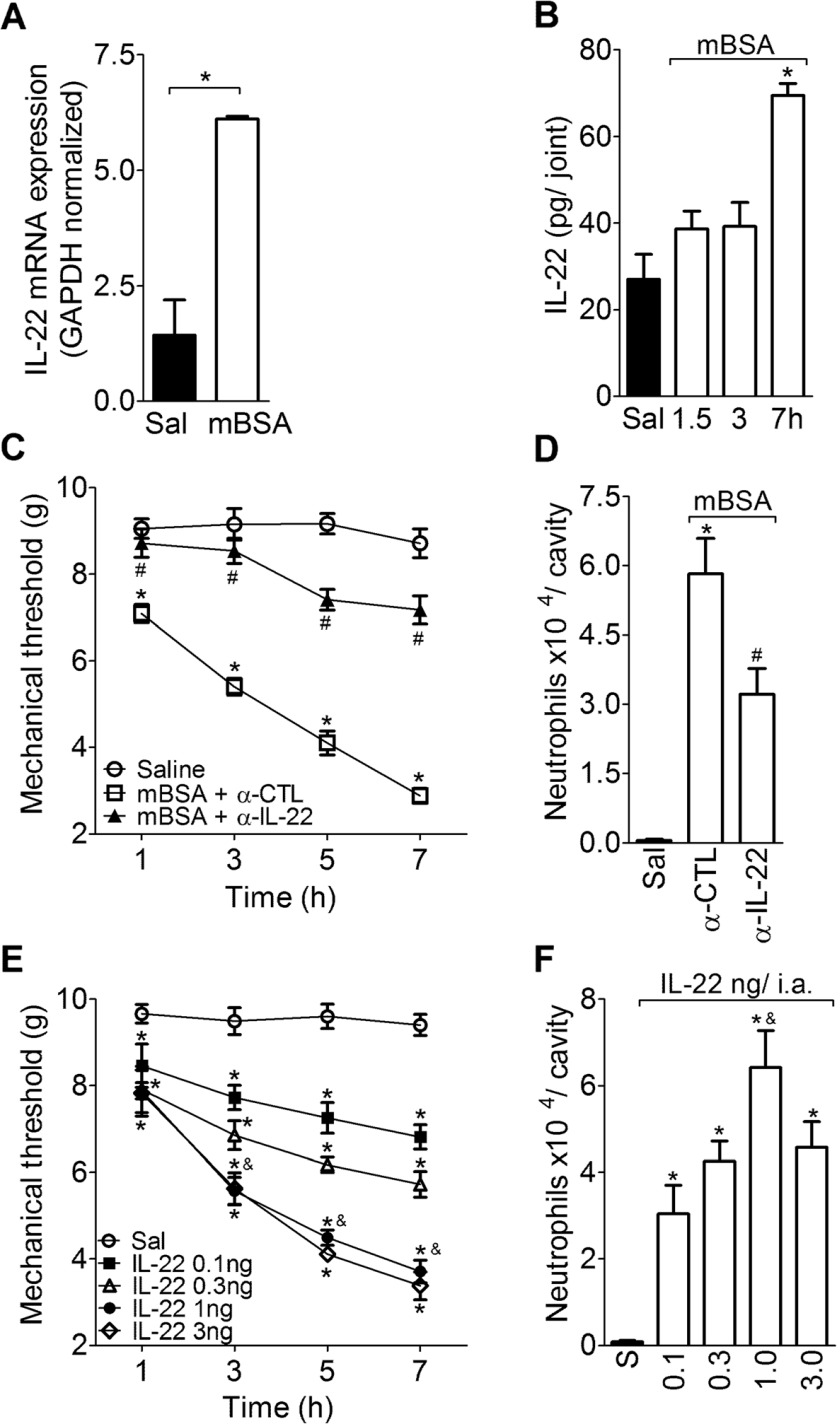
Role of IL-22 in the acute phase of AIA. **a** mBSA-immunized mice were challenged i.a. with 30 μg of mBSA or saline. After 3 h, synovial membranes were collected and analyzed for IL-22 mRNA expression by PCR. The gene expression was normalized to GAPDH expression. **b** The concentrations of IL-22 in the synovial membranes were determined at 1.5, 3 and 7 h after a challenge with either 30 μg of mBSA or saline in mBSA-immunized mice. **c** Articular hypernociception was evaluated 1–7 h after i.a. injection with either mBSA (30 μg) or saline in mBSA-immunized mice treated with a co-injection of IgG control (α-CTL) or α-IL-22 (5 μg/cavity) antibodies. **d** Neutrophil recruitment from the articular cavity 7 h after i.a. administration of mBSA (30 μg) or saline and treatment with a co-injection of α-CTL or α-IL-22 antibodies. **e** and **f** mBSA-immunized mice were challenged i.a. with either 0.1–3 ng of rmIL-22 or 10 μl of saline. **e** Articular hypernociception was evaluated over a period of 7 h. **f** Neutrophil recruitment from the articular cavity 7 h after i.a. injection of either IL-22 (0.1–3 ng per joint) or saline in mBSA-immunized mice. Data are the means ± SEM (*n* = 5). *P < 0.05, compared with the saline group; ^#^ *P* < 0.05, compared with the mBSA plus α-CTL group; and ^&^ *P* < 0.05, compared with the IL-22 0.1 and 0.3 ng i.a. groups. *AIA* antigen-induced arthritis, *GAPDH* glyceraldehyde 3-phosphate dehydrogenase, *i.a.* intra-articular, *Ig* immunoglobulin, *IL* interleukin, *mBSA* methylated bovine serum albumin, *PCR* polymerase chain reaction, *rmIL-22* recombinant murine IL-22

First, it was observed that the mBSA challenge into the femur-tibial joint of immunized mice induced an increase in IL-22 mRNA expression in synovial tissue 3 h after stimulus injection (Fig. 1a). Additionally, the i.a. injection of mBSA in immunized mice also promoted an increase in IL-22 protein levels in the synovial membrane at 7 h after the challenge (Fig. 1b). Local (joint) treatment of mice with a neutralizing antibody against IL-22 (injected simultaneously with mBSA) reduced joint nociception and neutrophil migration during arthritis development (Fig. 1c and d).

The local pro-inflammatory effect of IL-22 in AIA was further confirmed because a rmIL-22 challenge into the femur-tibial joint in mBSA-immunized mice induced a dose- and time-dependent decrease in the mechanical nociceptive threshold (Fig. 1e) and stimulation of neutrophil recruitment at 7 h after the challenge (Fig. 1f). A significant articular nociceptive response was observed 1 h after i.a. injections of IL-22 at all doses (0.1–3 ng), which progressively increased for 7 h following the challenge (Fig. 1e). An i.a. injection of IL-22 induced a significant dose-dependent (0.1–3 ng/joint) decrease in the nociceptive threshold (Fig. 1e) and an increase of neutrophil migration (Fig. 1f) when compared to mice that were injected with saline. A dose of 1 ng per joint of IL-22 produced a significant difference in articular pain and neutrophil recruitment compared with doses of 0.1 and 0.3 ng of IL-22. A dose of 3 ng/joint produced no further effects (Fig. 1e and f). Consequently, a dose of 1 ng per joint of IL-22 was used in subsequent experiments. The effects of IL-22 were reduced in SI mice when compared with mBSA-immunized mice (data not shown). In an attempt to exclude possible contamination with lipopolysaccharide (LPS) in the IL-22 preparations, IL-22 was administered into the joints of TLR4^−/−^ mice, and articular hypernociception and neutrophil migration were determined. It was observed that IL-22-induced articular hypernociception and neutrophil migration were similar in TLR4^−/−^ mice when compared with WT mice (Figure S1A and S1B in Additional file 1).

**Fig. 2:**
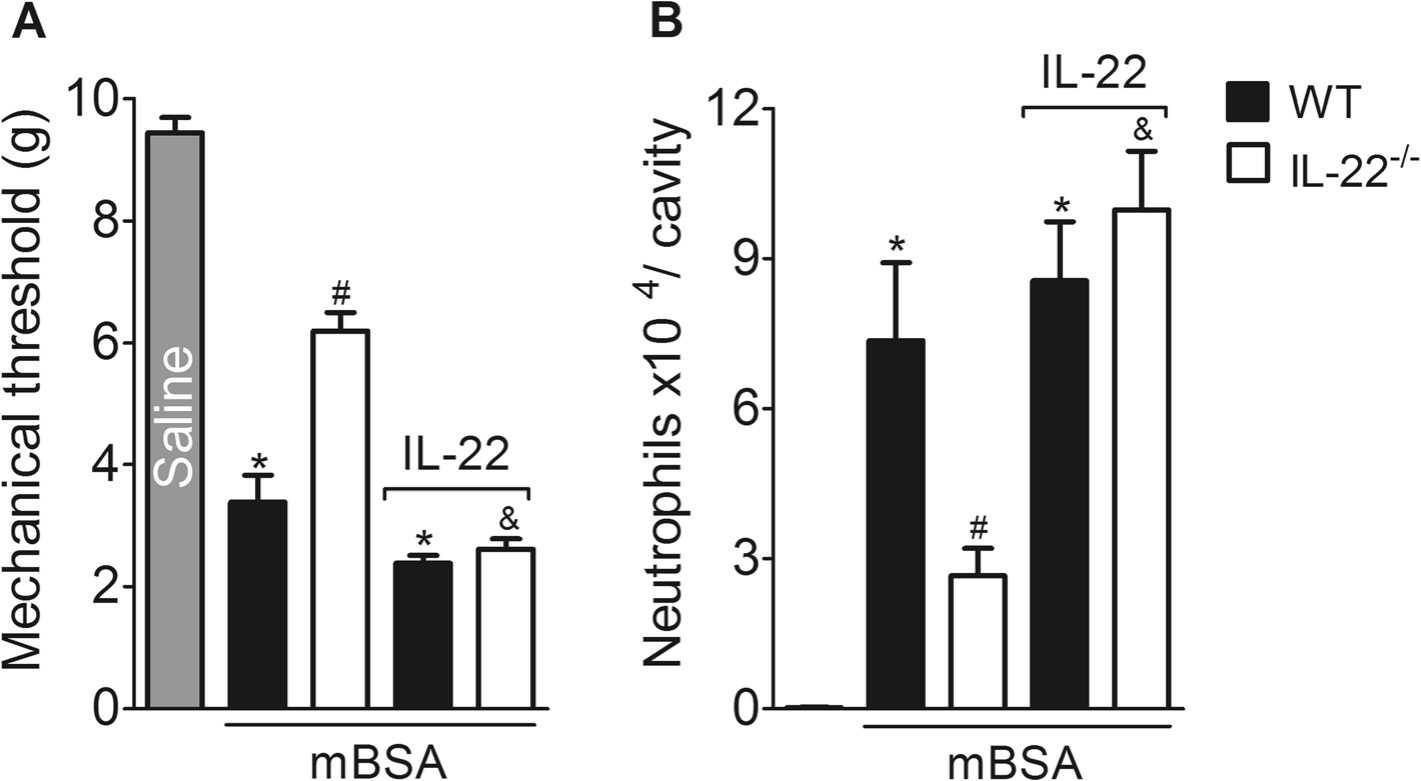
IL-22 is required for articular hypernociception and neutrophil migration in AIA. mBSA-immunized wild-type (WT) mice or IL-22^−/−^ mice were challenged i.a. with saline or mBSA (30 μg) and treated with a co-injection of rmIL-22 (0.3 ng) or vehicle. Articular hypernociception (**a**) and neutrophil migration (**b**) were evaluated 7 h following the challenge. Data are the means ± SEM (*n* = 5). *P < 0.05, compared with the saline group; ^#^ *P* < 0.05, compared with the WT mBSA group; and ^&^ *P* < 0.05 compared with the IL-22^−/−^ mBSA group. ^(*−/−*)^ deficient, *AIA* antigen-induced arthritis, *i.a.* intra-articular, *IL* interleukin, *mBSA* methylated bovine serum albumin, *rmIL-22* recombinant murine IL-22

To further confirm the participation of IL-22 in the pathophysiological mechanisms associated with AIA development, IL-22^−/−^ immunized mice were challenged with i.a injection of mBSA. On the one hand, corroborating with the pharmacological data, joint nociception and neutrophil migration were reduced in IL-22^−/−^ compared with WT mice 7 h after mBSA challenge (Fig. 2a and b). On the other hand, joint injections of exogenous rmIL-22 during the antigen challenge were able to reestablish articular hypernociception and neutrophil migration in IL-22^−/−^ mice (Fig. 2a and b).

**Fig. 3:**
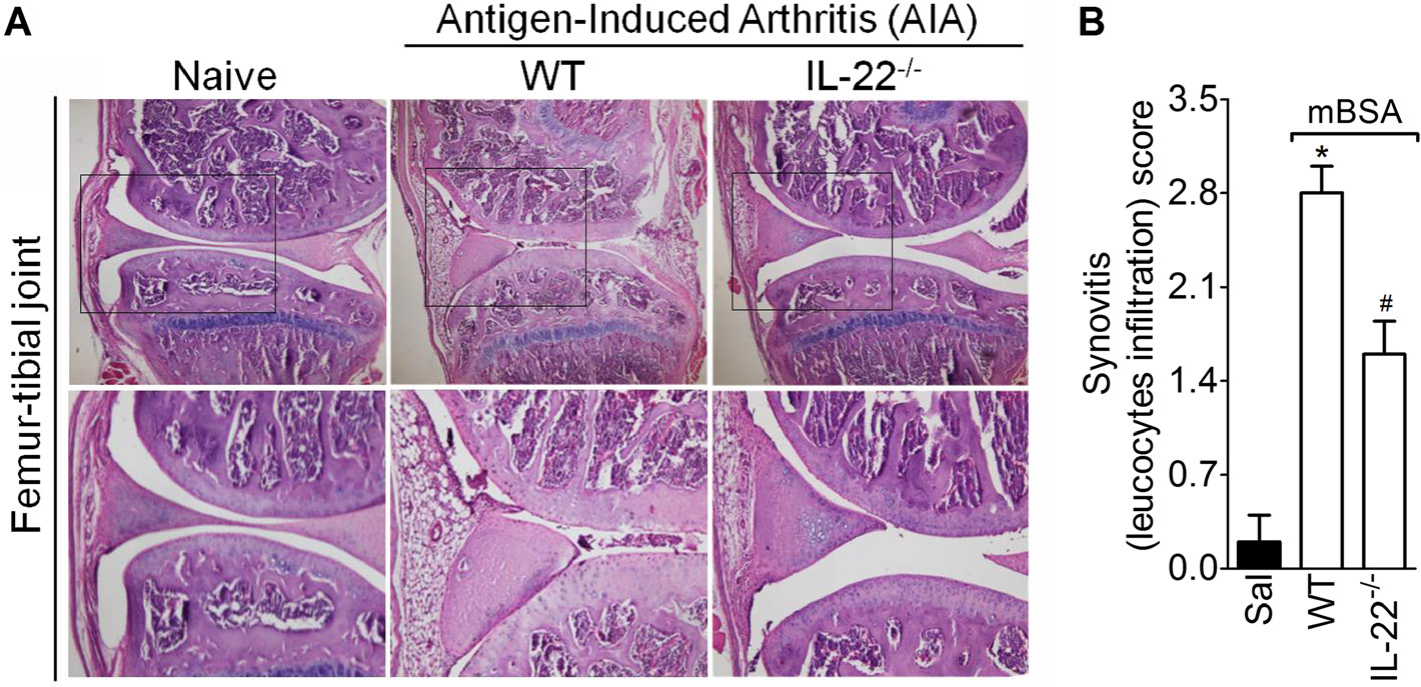
Lower histopathological score in IL-22-deficient mice than in WT mice with AIA. Histological analysis of knee joints of WT and IL-22^−/−^ immunized mice 7 h after mBSA (30 μg/i.a.) challenge. **a** Hematoxylin and eosin (H&E)-stained representative histological section (*upper panel*: magnification, 5×; and *lower panel*: higher magnification, 20×). **b** AIA severity was measured by the histological score for synovitis. The histological scoring system used was as follows: none (0), mild (1), moderate (2) or severe (3) synovitis. Data represent the mean ± SEM (*n* = 6), *P < 0.05, compared with the saline group; ^#^ *P* < 0.05, compared with the WT mBSA group. ^(*−/−*)^ deficient, *AIA* antigen-induced arthritis, *i.a.* intra-articular, *IL* interleukin, *mBSA* methylated bovine serum albumin, *WT* wild-type

Further exploring the participation of IL-22 in the acute inflammatory response during AIA, the histopathological analyses of joint tissue revealed that IL-22^−/−^ mice showed reduced synovitis with less cellular infiltration and the absence of synovial hyperplasia and cartilage damage when compared with WT mice 7 h after mBSA challenge (Fig. 3a and b). Altogether, these results suggest that local IL-22 production is required for the acute joint inflammatory response during AIA in mice.

To verify whether the reduced local inflammatory response in IL-22^−/−^ mice during AIA was due to an impaired immunization state, anti-mBSA antibody levels in the sera of immunized mice were determined. Interestingly, the serum levels of antibodies against mBSA, total IgG (Figure S2A in Additional file 2) and IgG2a (Figure S2B in Additional file 2) were similar in immunized WT and IL-22^−/−^ mice (Figure S2A and B in Additional file 2). Altogether, these findings suggest that the lower inflammatory response of IL-22^−/−^ mice during AIA was not associated with a decreased humoral immune response to mBSA.

Next, we developed a model of zymosan-induced arthritis in IL-22^−/−^ mice to evaluate whether the reduction of AIA in IL-22^−/−^ mice is a specific phenotype or a consequence of strain manipulation. Injection of zymosan (i.a., 30 μg/joint) induced mechanical articular hypernociception and intense neutrophil recruitment in WT mice that was not altered in IL-22^−/−^ mice (Figure S3A and B in Additional file 3). These findings argue against IL-22^−/−^ mice having a defect in the inflammatory response as a consequence of genetic manipulation.

### IL-22 mediates the acute inflammatory response of AIA through the modulation of IL-1β production

**Fig. 4:**
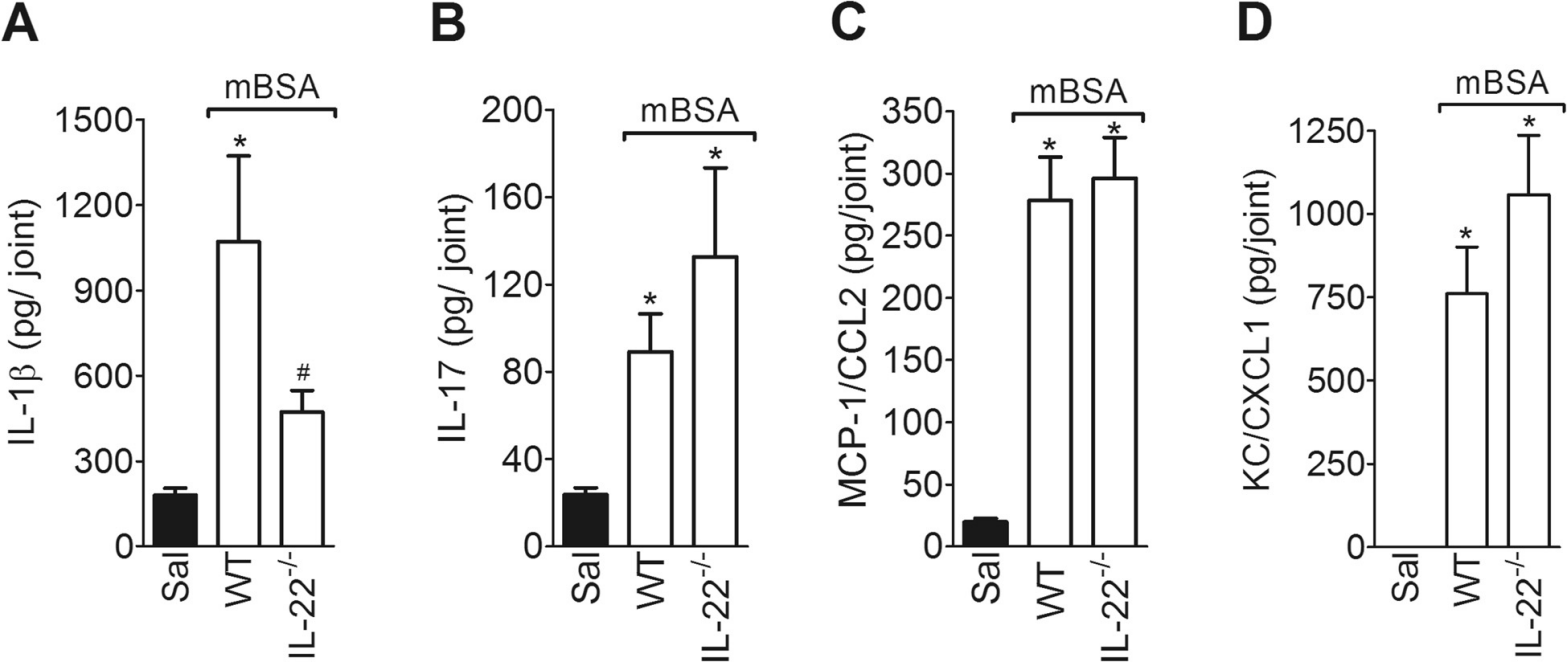
IL-22 differentially regulates local pro-inflammatory cytokines and chemokines in the early phase of AIA. The concentrations of IL-1β (**a**), IL-17 (**b**), MCP-1/CCL2 (**c**) and KC/CXCL1 (**d**) in the knee joint injected with mBSA (30 μg) or saline in WT and IL-22^−/−^ mBSA-immunized mice were determined at 3 h after challenge. The levels of cytokines and chemokines were evaluated by ELISA. Data are the means ± SEM (*n* = 5). *P < 0.05, compared with the saline group; and ^#^ *P* < 0.05, compared with the mBSA group. ^(*−/−*)^ deficient, *AIA* antigen-induced arthritis, *ELISA* enzyme-linked immunosorbent assay, *IL* interleukin, *KC/CXCL1* keratinocyte-derived chemokine, *mBSA* methylated bovine serum albumin, *MCP-1/CCL2* monocyte chemoattractant protein-1, *WT* wild-type

To identify the possible underlying mechanisms by which IL-22 mediates the local inflammatory response during AIA, local cytokine and chemokine production was determined in the joints of IL-22^−/−^ mice. The injection of mBSA into the joints in WT immunized mice increased the local production of IL-1β, IL-17, MCP-1/CCL2, and KC/CXCL1 at 3 h after the challenge (Fig. 4a–d, respectively). Interestingly, the production of IL-1β (Fig. 4a) was reduced in IL-22^−/−^ mice during the early phase of AIA, whereas the production of IL-17, MCP-1/CCL2 and KC/CXCL1 did not differ from WT mice (Fig. 4b–d).

**Fig. 5:**
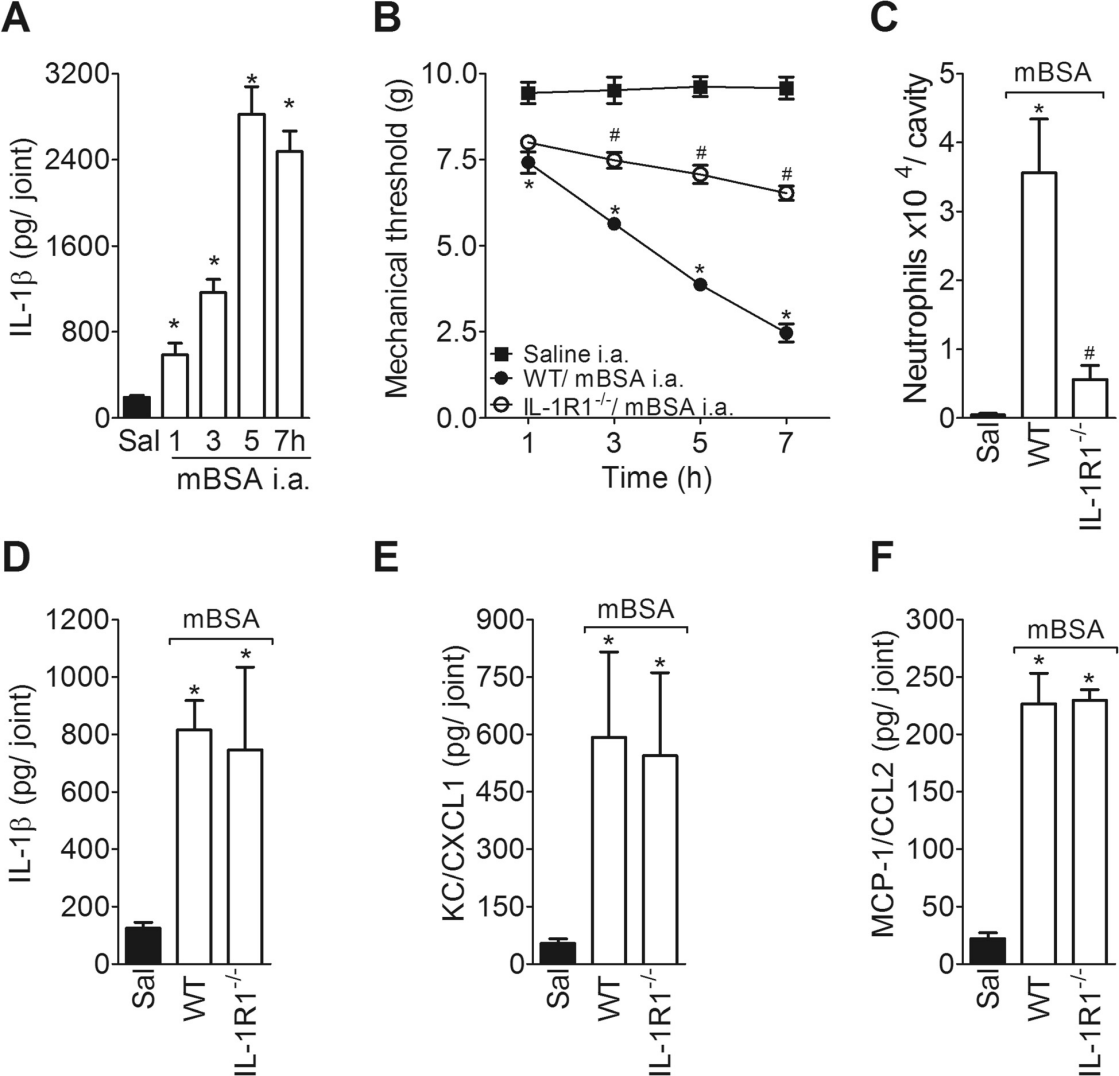
Participation of IL-1β in the development of AIA. **a** The concentrations of IL-1β in the knee joint injected with either 30 μg of mBSA or saline in mBSA-immunized mice were determined at 1, 3, 5 and 7 h after challenge. **b** Articular hypernociception was evaluated 1–7 h after i.a. injection with either mBSA (30 μg) or saline in mBSA-immunized WT or IL-1R1^−/−^ mice. **c** Neutrophil recruitment toward the articular cavity 7 h after i.a. administration of mBSA (30 μg) or saline in mBSA-immunized WT or IL-1R1^−/−^ mice. **d-f** The concentrations of IL-1β (**d**), KC/CXCL1 (**e**) and MCP-1/CCL2 (**f**) in the knee joint injected with mBSA (30 μg) or saline in WT and IL-1R1^−/−^ mBSA-immunized mice were determined at 3 h after challenge. Data are the means ± SEM (*n* = 5). *P < 0.05, compared with the saline group; and ^#^ *P* < 0.05, compared with the WT mBSA group. ^(*−/−*)^ deficient, *AIA* antigen-induced arthritis, *ELISA* enzyme-linked immunosorbent assay, *IL* interleukin, *KC/CXCL1* keratinocyte-derived chemokine, *mBSA* methylated bovine serum albumin, *MCP-1/CCL2* monocyte chemoattractant protein-1, *WT* wild-type

To confirm the participation of IL-1β in the inflammatory response during the early phase of AIA, IL-1R1^−/−^ mice were immunized, followed by i.a. injections of mBSA. First, mBSA challenge into the femur-tibial joint of WT mice produced a time-dependent increase in the levels of IL-1β in the joint tissues (Fig. 5a). Additionally, joint nociception and neutrophil migration during AIA were significantly diminished in IL-1R1^−/−^ mice after mBSA challenge compared with WT mice (Fig. 5b and c). The local production of IL-1β, KC/CXCL and MCP-1/CCL2, which were induced by i.a. administration of mBSA in WT immunized mice, was not altered in IL-1R1^−/−^ mice (Fig. 5d–f).

**Fig. 6:**
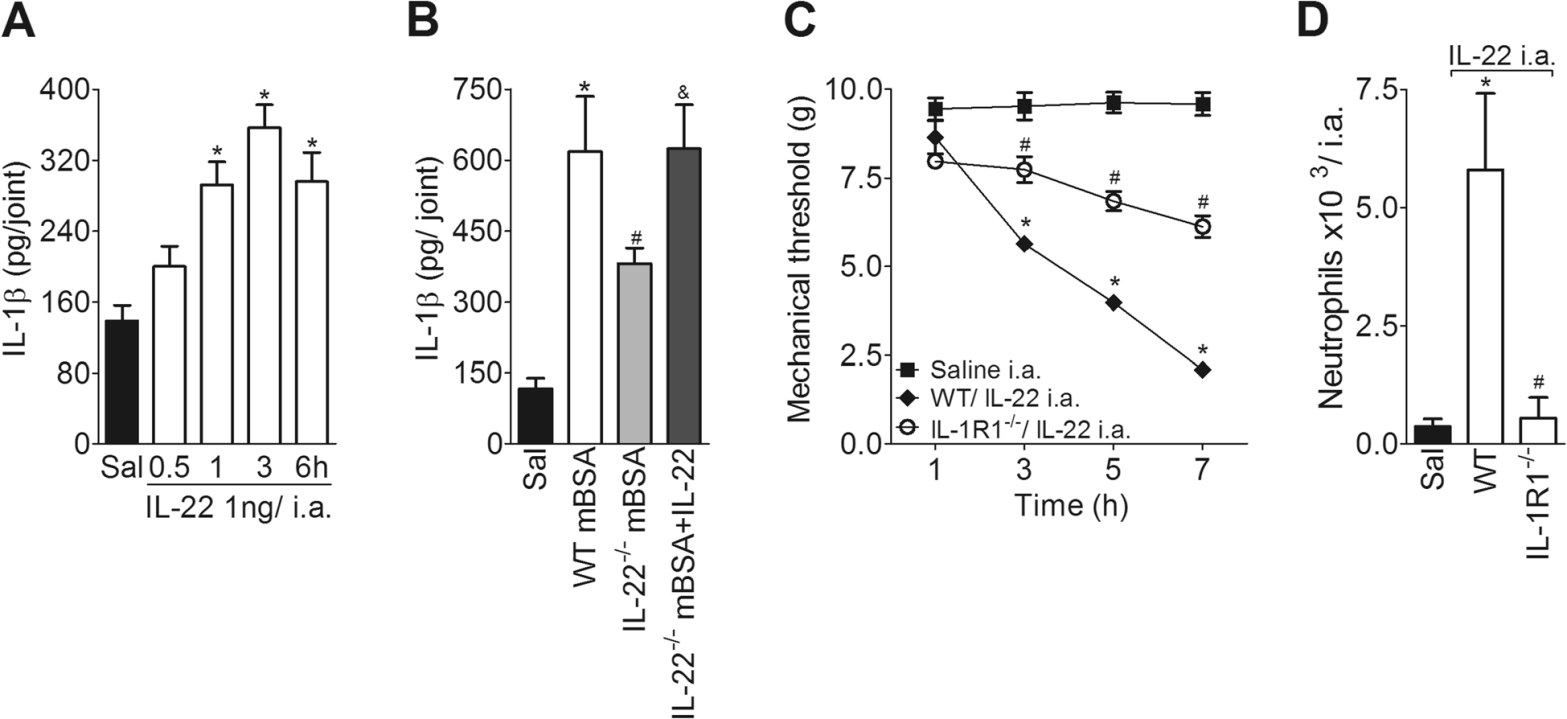
IL-1β mediates the pro-inflammatory effects of IL-22 in AIA. **a** mBSA-immunized mice were injected i.a. with IL-22 (1 ng) or saline and the concentrations of IL-1β were determined at 0.5, 1, 3 and 6 h after challenge. **b** mBSA-immunized WT or IL-22^−/−^ mice were challenged i.a. with mBSA (30 μg) or saline and treated with a co-injection of IL-22 (0.3 ng) or vehicle. The concentrations of IL-1β were determined 3 h after challenge. **c** and **d** mBSA-immunized WT or IL-1R1^−/−^ mice were challenged i.a. with IL-22 (1 ng per joint) or saline and articular hypernociception (**c**) and neutrophil migration (**d**) were evaluated 7 h following the challenge. Data are expressed as the means ± SEM (*n* = 5). *P < 0.05, compared with the saline group; ^#^ *P* < 0.05 compared the WT mBSA or WT IL-22 group; and ^&^ *P* < 0.05 compared with the IL-22^−/−^ mBSA group. ^(−/−)^, deficient, *AIA* antigen-induced arthritis, *i.a.* intra-articular, *IL* interleukin, *mBSA* methylated bovine serum albumin, *WT* wild-type

According to the hypothesis that IL-22 participates in the joint inflammatory response during AIA through the stimulation of IL-1β production, the injection of IL-22 into the joints of mBSA-immunized mice significantly increased the levels of IL-1β in a time-dependent manner (Fig. 6a). Moreover, the injection of exogenous rmIL-22 into the joints of IL-22^−/−^ mice reestablished the levels of IL-1β compared with the levels of WT mice during the mBSA challenge (Fig. 6b). In addition, joint hypernociception and neutrophil recruitment induced by i.a. injection of rmIL-22 were reduced in IL-1R1^−/−^ mice (Fig. 6c and d). Taken together, these data indicate that IL-22 might have a modulatory role in the production of IL-1β during the early phase of AIA that accounts for the local inflammatory response.

In an attempt to evaluate the mechanism involved in the production of IL-1β induced by IL-22 during the acute phase of AIA, we first pretreated WT immunized mice with fucoidin (a leukocyte adhesion inhibitor) [32], and 3 h after a challenge with rmIL-22 (1 ng per joint), the levels of IL-1β were determined by ELISA. Interestingly, the joint production of IL-1β induced by IL-22 was significantly increased in WT immunized mice pretreated with fucoidin when compared with the vehicle group (Figure S4A in Additional file 4), suggesting that neutrophils that are recruited to the joint after IL-22 administration are not the main source of IL-1β. By further investigating the mechanisms involved in the production of IL-1β triggered by IL-22, it was observed that articular hypernociception and neutrophil migration induced by i.a. injections of IL-22 in WT immunized mice were significantly diminished in ASC^−/−^ immunized mice 7 h after the challenge (Figure S4B and C in Additional file 4). These results suggest (although indirectly) that IL-22 stimulation of joint IL-1β production in the acute phase of AIA might be dependent on ASC.

## Discussion

It is well accepted that the presence of various pro-inflammatory cytokines in the joint environment contributes to the pathophysiology of autoimmune arthritis [33]. In the last decade, IL-22, a cytokine with pleiotropic effects, has been implicated in joint pathology during inflammatory arthritis [22, 23]. For instance, the presence of IL-22 and IL-22R1 in synovial tissue [22, 34] and elevated levels of this cytokine in the serum of RA patients have been demonstrated [35, 36]. However, the role of IL-22 in the pathophysiology of RA remains under debate. In fact, experimental evidence presents contradictory effects (protective *versus* pathogenic) for IL-22 in the genesis of arthritis [26, 27, 37]. Herein, we demonstrated that IL-22 is pathogenic in the early phase of AIA in mice, mediating neutrophil migration and pain. IL-22 seems to be important in the induction of the local production of IL-1β, which is an important pro-arthritic cytokine that mediates leukocyte recruitment to the joint as well as the joint pain.

Previous reports have observed an increase in IL-22 expression in experimental models of arthritis in mice. Indeed, Geboes and collaborators (2009) [26] first demonstrated that IL-22 and IL-22R1 levels were elevated in the serum, lymphoid tissue and others tissues in immunized mice during collagen-induced arthritis (CIA). In addition, in the experimental model of spontaneous arthritis observed in IL-1 receptor antagonist (Ra) ^−/−^ mice, the level of IL-22 was shown to be upregulated in inflamed synovial tissue [38]. Moreover, CD4^+^T cells isolated from arthritic joints and splenocytes express IL-22 after the induction of AIA, suggesting the possible involvement of IL-22 in this model of arthritis [39, 40]. In further support of this suggestion, in our experiments, we observed that mRNA expression and protein levels of IL-22 in the synovial membrane increased during the onset of AIA. Moreover, the results obtained using pharmacological (antibody against IL-22) and genetic (IL-22^−/−^ mice) inhibition of this cytokine indicate a pro-inflammatory role of IL-22 in the earlier phase of AIA. Supporting these findings, the direct administration of recombinant murine IL-22 into the joints recapitulates the clinical (pain enhancement) and morphological (neutrophil migration) characteristics of arthritis. In agreement with our results, treatment with anti-IL-22 reduced inflammation and bone erosion in IL-1-driven arthritis [38]. Furthermore, neutralization of IL-22 after the onset of CIA diminished the severity of arthritis [37].

On the other hand, a recent report showed that IL-22^−/−^ mice present no differences in knee swelling and macroscopic inflammation scores (evaluated by redness) compared to WT mice 7 days after AIA induction [41]. This lack of effect can be explained by the intense amount of stimuli (mBSA- 60 μg/i.a.) administered in the joint compared to that used in our study (mBSA- 30 μg/i.a.). Furthermore, the time of evaluation of the parameters was also different. While we analyzed the role of IL-22 in the acute phase of AIA (7 h after induction), van Hamburg and colleagues (2013) evaluated the inflammatory parameters 7 days after AIA induction. Thus, we could not exclude that IL-22 might have a different role in acute and late phases of the disease. In fact, there is evidence that IL-22 has a dual role in inflammatory diseases [42, 43].

The experimental model of antigen (mBSA)-induced arthritis is a suitable and reproducible experimental system that exhibits several features with histopathological findings similar to those observed in human RA [28]. The adaptive immune response in AIA is dependent on antigen-specific CD4^+^ T cells and their derived mediators and, to a lesser extent, is mediated by serum antibodies [44, 45]. Interestingly, the titers of anti-mBSA antibodies were similar in WT and IL-22^−/−^ mice. This is noteworthy, because IL-22^−/−^ mice exhibit higher levels of collagen type II-specific and total IgG antibodies during CIA [26]. Although anti-mBSA antibodies have been detected in AIA, it is important to mention that this RA model is less dependent on the humoral response [28]. Indeed, B-lymphocyte-deficient μMT/μMT mice (which lack mature B lymphocytes and do not produce IgM or IgG antibodies) develop AIA that is similar to that developed in WT mice, suggesting that B cells do not participate, at least during the acute phase of AIA [46]. Besides the humoral response, it was demonstrated that the proliferative response, T cell phenotype and cytokine levels in the supernatant of lymph node cells were similar between arthritic IL-22^−/−^ and WT mice [41]. Taken together, these pieces of evidence indicate that the lower inflammatory response of IL-22^−/−^ mice during AIA was not associated with a decreased humoral or cellular immune response to mBSA.

Previous reports have shown that mBSA injection induces the production of several cytokines (IL-17, IL-1β) and chemokines (CXCL1/KC) during antigen-induced inflammation [9, 29, 47]. Interestingly, our findings demonstrate that only IL-1β production was decreased by the genetic inhibition of IL-22, indicating that the pathogenic role of IL-22 during AIA onset could be mediated through IL-1β. In accordance with our results, it was demonstrated that the increased IL-1β expression in synovial tissues during CIA was diminished in IL-22^−/−^ mice [26]. In addition, in an experimental model of skin inflammation, the levels of IL-1β were decreased in IL-22^−/−^ mice [48]. Nevertheless, we cannot exclude the involvement of other mediators in this effect of IL-22 in the early phase of AIA.

The participation of IL-1β was also observed in other models of articular inflammation. In fact, it was shown that IL-1β mediates inflammation and dysfunction of the joint induced by the injection of monosodium urate (MSU) crystals into the knee in an experimental model of gout [49]. Additionally, in the spontaneous inflammatory arthritis model of K/BxN T cell receptor-transgenic mice, IL-1β is absolutely necessary for joint inflammation [50]. Our results indicate that IL-1β mediates the induction of inflammatory events (neutrophil migration and pain) in AIA, which is not dependent on the modulation of the production of cytokines and chemokines. Thus, it is possible that the pro-inflammatory effects of IL-1β that are observed during AIA might be directly connected to pain induction as a consequence of the stimulation of the production of prostaglandins (which are important in the sensitization of primary nociceptive fibers) and on neutrophil migration, since it can modulate endothelial adhesion molecules [51–53]. In addition, it is important to note that IL-1β also has direct and indirect effects in the nociceptive neurons, inducing their sensitization and neutrophil migration [8, 54–56]. In this context, our group demonstrated that IL-1β mediates neutrophil migration induced by several cytokines, including IL-17, during AIA [9]. In the present study, no difference in the production of IL-17A was observed between IL-22^−/−^ and WT mice, suggesting that the pro-arthritic role of IL-22 appears to be independent of IL-17 release in the joint. It is important to propose that IL-17 can induce IL-22 or that these cytokines may act together to mediate the pathogenesis of AIA. For instance, the interdependence of IL-17 and IL-22 in other models of experimental arthritis has been suggested [38, 57]. Furthermore, the correlation of IL-17 and IL-22 was also observed in patients with arthritis [34]. Interestingly, the synergistic effect of these cytokines has already been demonstrated in other models of inflammation. Indeed, in an experimental model of airway inflammation, it was suggested that IL-22 can act cooperatively with IL-17A, which contributes to the pathological role of IL-22 in the lung [42, 43].

Concerning the mechanism involved in the IL-22-induced production of IL-1β, our data indicate that it is not dependent on recruited neutrophils, but it may be dependent on resident cells. Another important observation is that the pro-inflammatory role of IL-22 in joint inflammation appears to be dependent on ASC. In accordance, it was demonstrated that the adapter molecule ASC mediates the pro-inflammatory response in AIA and production of IL-1β [58]. However, it appears that the role of ASC in AIA development does not require NLRP3/caspase-1 and NLRC4/caspase-1 inflammasomes [58]. Thus, further studies are necessary to elucidate the relationship between IL-22 and ASC in the development of AIA.

## Conclusions

In summary, the present data provides evidence that joint IL-22 is pathogenic during the onset of antigen-induced arthritis in mice. Our findings also suggest that the pro-inflammatory role of IL-22 in the physiopathology of arthritis is dependent on the modulation of IL-1β production in the joint, which may be dependent on ASC. Therefore, it seems that IL-22 is a possible therapeutic target in the initial phase of RA, controlling the various symptoms present in arthritis.

## Additional files

Additional file 1: Figure S1. Pro-inflammatory effects of IL-22 in TLR4^−/−^ mice are similar to WT mice. (A and B) WT or TLR4^−/−^ mice were challenged i.a. with IL-22 (1 ng per joint) or saline, and articular hypernociception (A) and neutrophil migration (B) were evaluated 7 h after the challenge. Data are presented as the means ± SEM (*n* = 5). ^*^ *P* < 0.05, compared with the saline group. (TIFF 1072 kb) Additional file 2: Figure S2. Immunoglobulin production of WT and IL-22^−/−^ mice. Levels of total IgG (A) and IgG2a (B) antibodies against mBSA in the sera of WT and IL-22^−/−^ mBSA-immunized mice. Data are the means ± SEM (*n* = 5). (TIFF 1185 kb) Additional file 3: Figure S3. Role of IL-22 in zymosan-induced arthritis. (A and B) WT or IL-22^−/−^ mice were challenged i.a. with zymosan (30 μg per joint) or saline and articular hypernociception (A) and neutrophil migration (B) were evaluated 7 h after the challenge. Data are presented as the means ± SEM (*n* = 5). ^*^ *P* < 0.05, compared with the saline group. (TIFF 1105 kb) Additional file 4: Figure S4. The inflammasome adapter ASC participates in articular hypernociception and neutrophil migration that are induced by IL-22 during AIA. (A) The concentrations of IL-1β in the knee joint injected with 1 ng of IL-22 or saline and pretreated with fucoidin (20 mg/kg, i.v. 15 min before stimuli injection) in mBSA-immunized mice were determined 3 h after the challenge. (B) Articular hypernociception was evaluated 1–7 h after i.a. injection with either IL-22 (1 ng per joint) or saline in mBSA-immunized WT or ASC^−/−^ mice. (C) Neutrophil recruitment toward the articular cavity 7 h after i.a. administration of IL-22 (1 ng) or saline in mBSA-immunized WT or ASC^−/−^ mice. Data are presented as the means ± SEM (*n* = 5). ^*^ *P* < 0.05, compared with the saline group; and ^#^ *P* < 0.05, compared with the vehicle (−)/WT IL-22 groups. (TIFF 930 kb)

## Abbreviations

^(−/−)^: deficient/knockout mice
AIA: antigen-induced arthritis
ASC: apoptosis-associated speck-like protein containing a C-terminal caspase recruitment domain
CFA: complete Freund adjuvant
CIA: collagen-induced arthritis
CT: cycle threshold
CTL: control
EDTA: ethylenediaminetetraacetic acid
ELISA: enzyme-linked immunosorbent assay
g: grams
GAPDH: glyceraldehyde 3-phosphate dehydrogenase
H&E: hematoxylin and eosin
i.a.: intra-articular
Ig: immunoglobulin
IL: interleukin
KC/CXCL1: keratinocyte-derived chemokine
LPS: lipopolysaccharide
LTi: lymphoid tissue inducer
mBSA: methylated bovine serum albumin
MCP-1/CCL2: monocyte chemoattractant protein-1
MMPs: matrix metalloproteinases
MSU: monosodium urate
NK: natural killer
PBS: phosphate-buffered saline
Ra: receptor antagonist
RA: rheumatoid arthritis
rmIL-22: recombinant murine IL-22
RT-PCR: reverse transcription-polymerase chain reaction
s.c.: subcutaneous
SI: sham-immunized
Th: T helper
TLR4: Toll-like receptor 4
TNF: tumor necrosis factor
WT: wild-type
ZIA: zymosan-induced arthritis
