# Supplementary figures and images for: Joint production of IL-22 participates in the initial phase of antigen-induced arthritis through IL-1β production
Source: Arthritis Res Ther. 2015 Sep 2;17(1):235. doi: 10.1186/s13075-015-0759-2 (PMC4556214; doi:10.1186/s13075-015-0759-2)

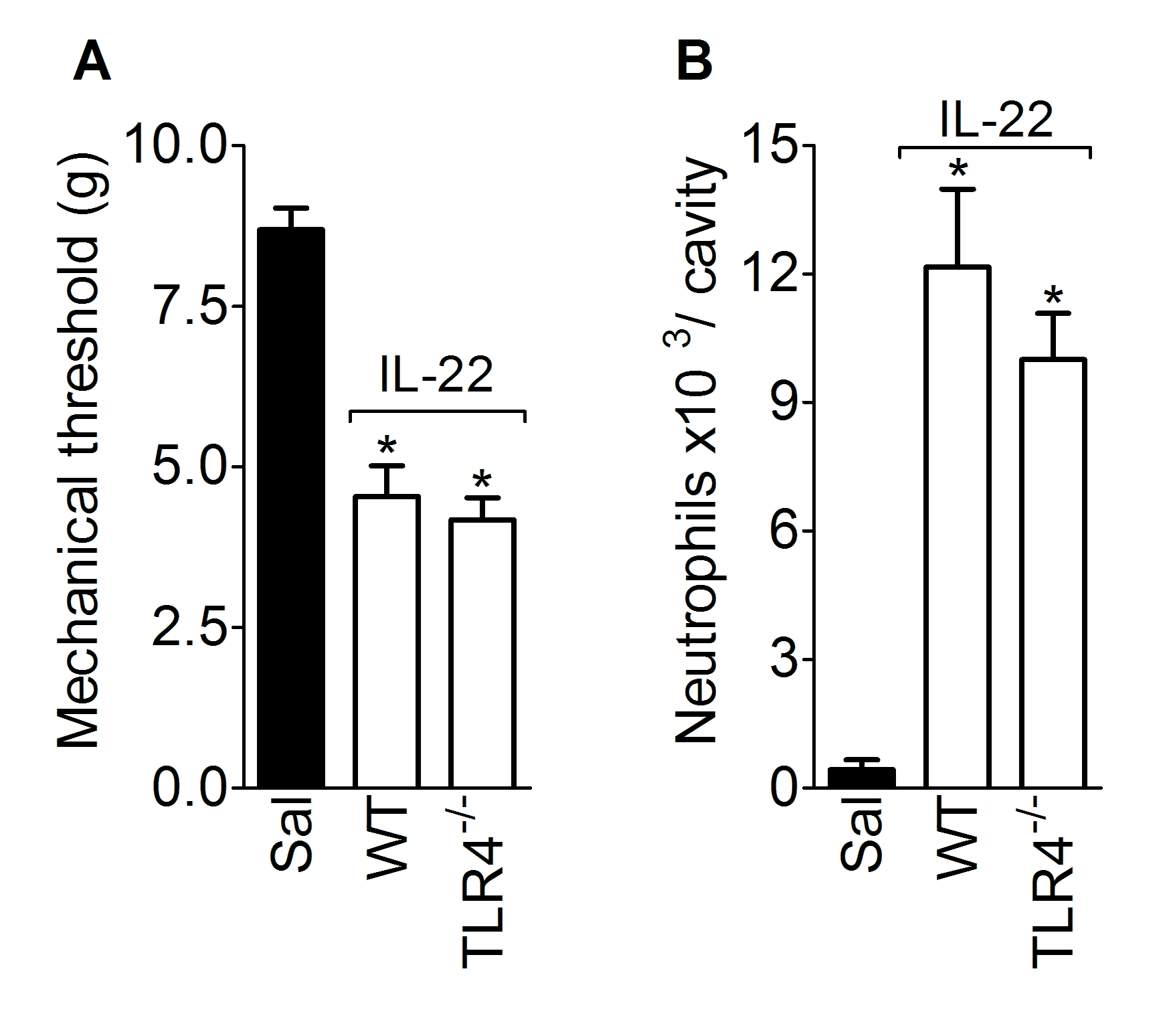

Supplement: Additional file 1: Figure S1. — Pro-inflammatory effects of IL-22 in TLR4−/− mice are similar to WT mice. (A and B) WT or TLR4−/− mice were challenged i.a. with IL-22 (1 ng per joint) or saline, and articular hypernociception (A) and neutrophil migration (B) were evaluated 7 h after the challenge. Data are presented as the means ± SEM (n = 5). * P < 0.05, compared with the saline group. (TIFF 1072 kb) [file 13075_2015_759_MOESM1_ESM.tiff]

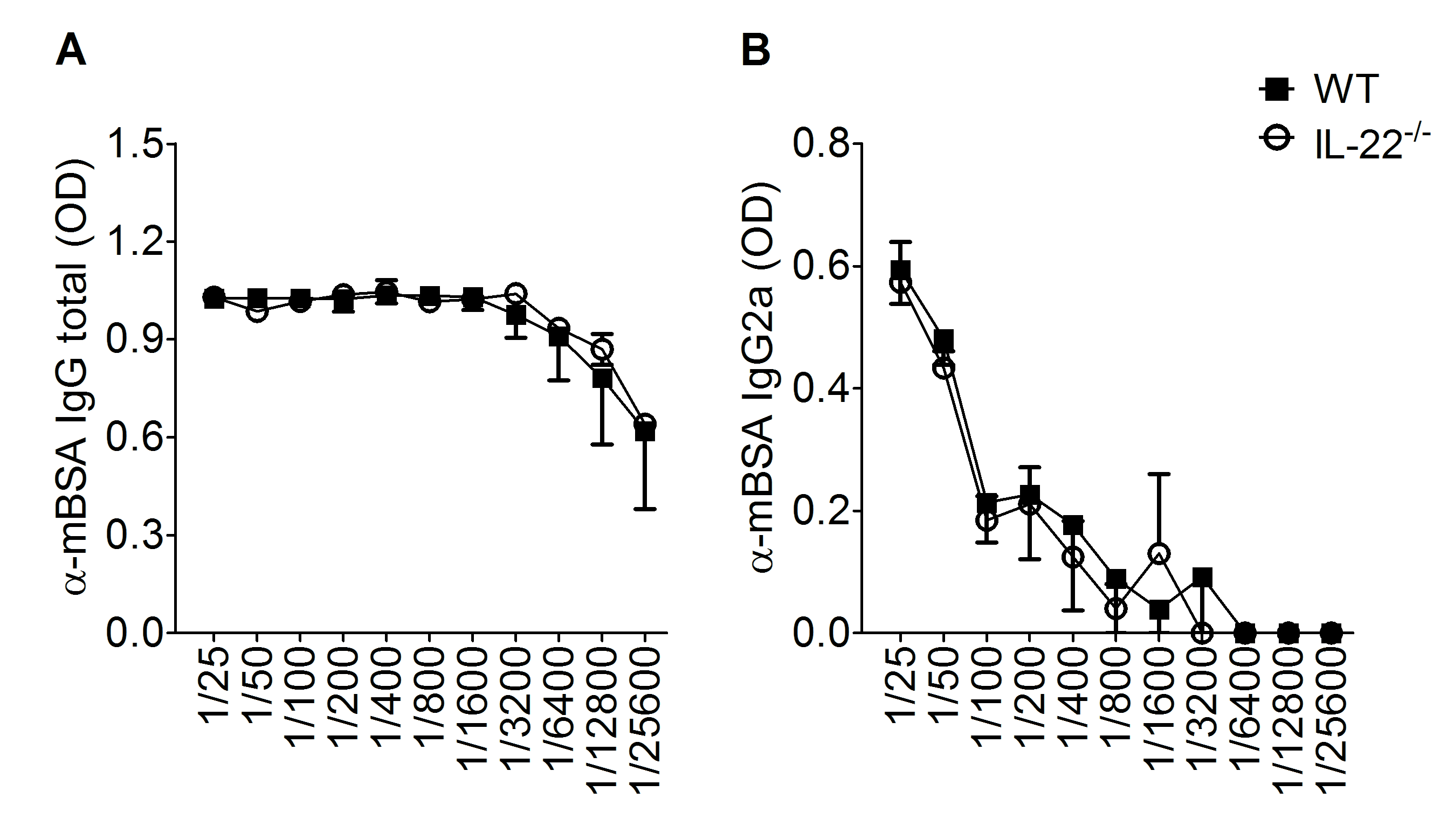

Supplement: Additional file 2: Figure S2. — Immunoglobulin production of WT and IL-22−/− mice. Levels of total IgG (A) and IgG2a (B) antibodies against mBSA in the sera of WT and IL-22−/− mBSA-immunized mice. Data are the means ± SEM (n = 5). (TIFF 1185 kb) [file 13075_2015_759_MOESM2_ESM.tiff]

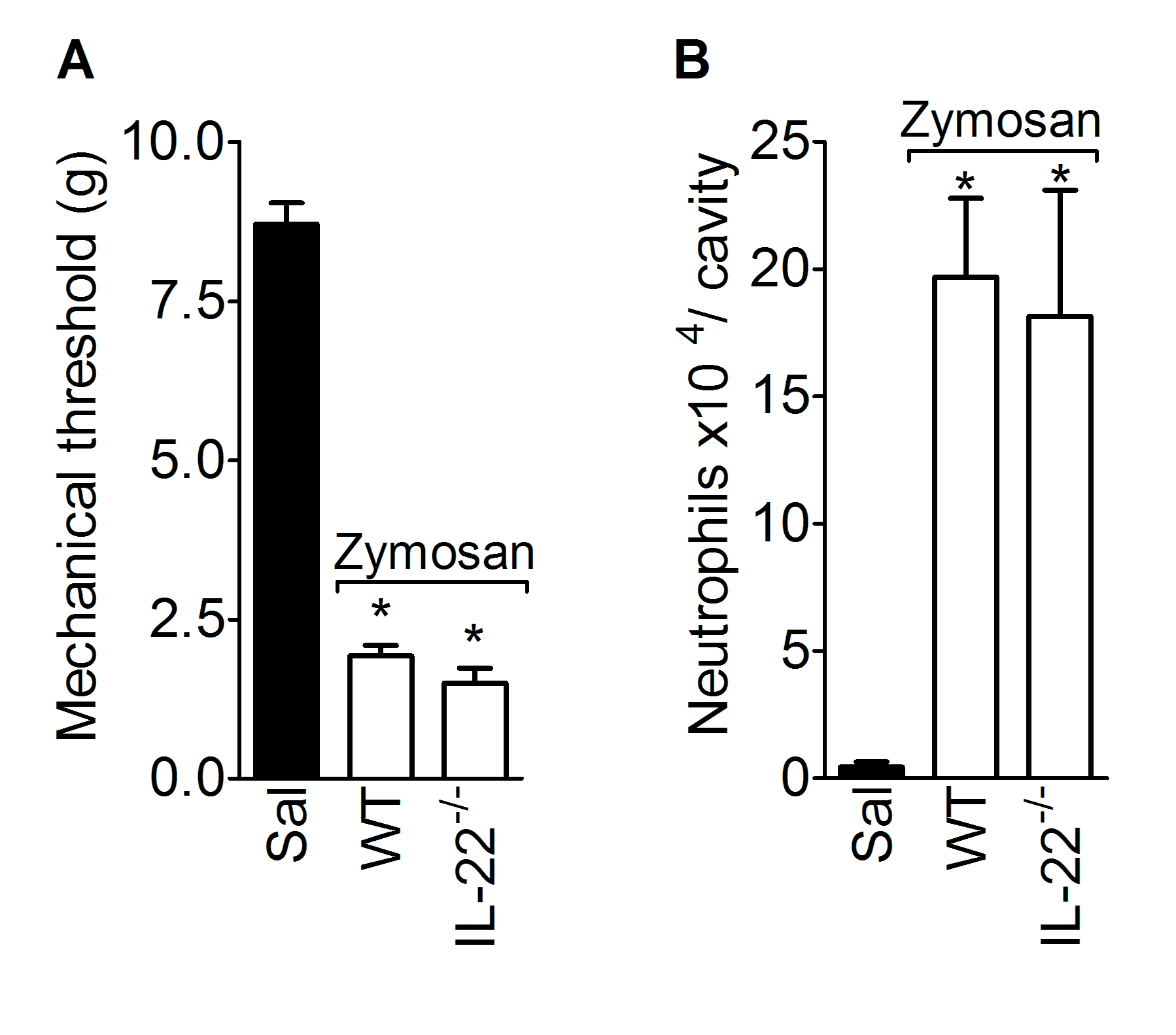

Supplement: Additional file 3: Figure S3. — Role of IL-22 in zymosan-induced arthritis. (A and B) WT or IL-22−/− mice were challenged i.a. with zymosan (30 μg per joint) or saline and articular hypernociception (A) and neutrophil migration (B) were evaluated 7 h after the challenge. Data are presented as the means ± SEM (n = 5). * P < 0.05, compared with the saline group. (TIFF 1105 kb) [file 13075_2015_759_MOESM3_ESM.tiff]

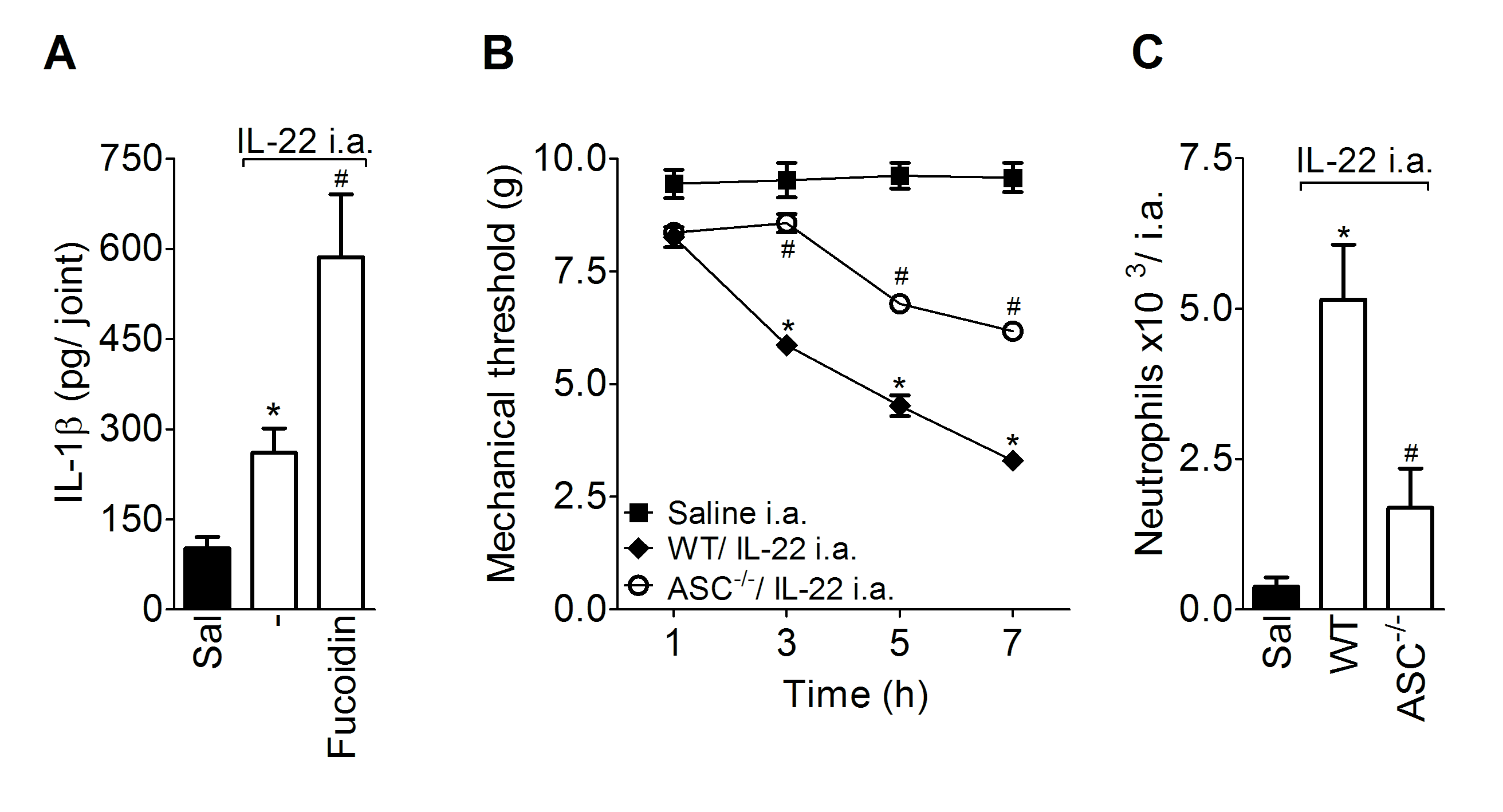

Supplement: Additional file 4: Figure S4. — The inflammasome adapter ASC participates in articular hypernociception and neutrophil migration that are induced by IL-22 during AIA. (A) The concentrations of IL-1β in the knee joint injected with 1 ng of IL-22 or saline and pretreated with fucoidin (20 mg/kg, i.v. 15 min before stimuli injection) in mBSA-immunized mice were determined 3 h after the challenge. (B) Articular hypernociception was evaluated 1–7 h after i.a. injection with either IL-22 (1 ng per joint) or saline in mBSA-immunized WT or ASC−/− mice. (C) Neutrophil recruitment toward the articular cavity 7 h after i.a. administration of IL-22 (1 ng) or saline in mBSA-immunized WT or ASC−/− mice. Data are presented as the means ± SEM (n = 5). * P < 0.05, compared with the saline group; and # P < 0.05, compared with the vehicle (−)/WT IL-22 groups. (TIFF 930 kb) [file 13075_2015_759_MOESM4_ESM.tiff]
